# HEARING AND OLFACTORY LOSS ASSOCIATED WITH ALTERED BRAIN STRUCTURE AND CONNECTIVITY IN DEMENTIA RISK

**DOI:** 10.1093/geroni/igae098.1996

**Published:** 2024-12-31

**Authors:** Natalie Phillips, Tristin Best, Nicole Grant, Ameera Kabir, M Kathleen Pichora-Fuller, Jennifer Campos, Paul Mick, Walter Wittich

**Affiliations:** Concordia University, Montréal, Quebec, Canada; Concordia University, Montréal, Quebec, Canada; Concordia University, Montréal, Quebec, Canada; Concordia University, Montréal, Quebec, Canada; University of Toronto, Toronto, Ontario, Canada; The KITE Research Institute, University Health Network, Toronto, Ontario, Canada; University of Saskatchewan, Saskatoon, Saskatchewan, Canada; University of Montréal, Montréal, Quebec, Canada

## Abstract

Sensory loss in hearing, vision, and olfaction is highly prevalent in older adults and each is associated with higher risk of developing dementia. We sought to identify whether these sensory factors are associated with alterations in brain function and structure in older adults with or at risk for dementia. Our groups included those with low risk (normal cognition, no cognitive complaints (NC=128; mean age=69 years; mean education=16 years)), and those with higher risk, namely 135 individuals with subjective reports of cognitive decline (SCD) but normal cognition (age=70; education=17), and 241 participants with mild cognitive impairment (MCI; age=72; education=16), and 93 with Alzheimer’s disease (AD, age=75; education=15). We used data from the COMPASS-ND study (Release 7). Hearing loss was assessed with pure-tone screening, vision with contrast sensitivity, and olfaction with the Brief Smell Identification Test. Normal sensory performance was observed in a minority of participants and decreased across the dementia risk spectrum groups (e.g., NC: 29%; SCD: 29%; MCI: 11%; AD: 2%). Olfactory deficits were the most frequent, followed by hearing impairment, and then deficits in visual contrast sensitivity. Deficits in multiple sensory domains were highly prevalent, with 37% of participants with AD having deficits in two or more domains. Preliminary analyses indicate that hearing loss is associated with altered connectivity in the default mode network and olfactory loss is associated with reduced hippocampal volumes in SCD. Thus, sensory loss is highly prevalent and co-morbid in persons with or at risk for dementia and has implications for cognition and brain function.

